# The Book World of Medicine and Science

**Published:** 1894-12-01

**Authors:** 


### THE BOOK WORLD OF MEDICINE AND SCIENCE.

The Treatment of Wounds, Ulcers, and Abscesses. By W. Watson Cheyne, M.B. Ed., F.R.S., F.R.C.S. (Edinburgh and London: Young J. Pentland. 1894. pp. 188.)

Although this is but a small book it is one of considerable importance, dealing, as it does, not merely with the theory of antiseptic surgery, but with the results of a large practical experience. The full faith which possesses the author is expressed in the following extract from the preface: "Suppuration occurring in a wound made through unbroken skin indicates that the surgeon has committed some avoidable error in his methods, unless indeed the wound is in the immediate neighbourhood of or communicates with some mucous canal; it will not do with our present knowledge and experience to attribute such an occurrence to constitutional defects, to bad materials (for, after all, the surgeon is responsible for the purity of the materials which he uses), &c., it is evident that some error has crept into the manipulations, and it is only by honestly acknowledging this to one's self and by searching for the fault that such an occurrence can be avoided on another occasion."

The careful and complete description of the methods of antiseptic work are altogether admirable, and one cannot but be struck with the comparative simplicity of many of the means employed.

Professor Cheyne lays strong emphasis on the uselessness of employing antiseptic solutions to granulating surfaces, or for the purpose of washing out septic granulating wounds. The organisms are situated in the surface tissue itself and are not got at by mere antiseptic lotions.

In regard to the treatment of compound fractures the most painstaking care is advised, not merely as to skin cleanliness, but as to the complete cleansing and disinfection of the inner recesses of the wound. Of course, an anæsthetic is required and the parts must be thoroughly exposed. "I believe," says the author, "that in most instances of compound fracture it is best to sponge the whole of the exposed surface of the tissues, both bone and soft tissues, with undiluted carbolic acid. This method is much more certain than the injection of the weaker solutions, and, as was shown by Sir Joseph Lister's earlier work, and as is now our experience, it does not complicate the subsequent healing of the wound, seeing that union by first intention is not aimed at."

In ordinary lacerated wounds also, which have become infiltrated with dirt, this sponging with the strong acid is also advised, especially where earth has entered, and where, therefore, there is considerable risk of tetanus.

The chapters on the treatment of abscesses and of ulcers are full of interest, and will repay careful perusal. No doubt to those unused to it the application of antiseptic methods is in some ways tiresome, but after awhile a form of antiseptic automatism becomes developed, so that the surgeon tends to do what is right without conscious thought, and when that has once been attained there should be no difficulty and but little trouble, while on the other hand there will always be endless satisfaction in doing perfect work. The book is one which should be carefully studied by all who are engaged, even only occasionally, in surgical work.

### A NEW CHURCH MAGAZINE.

Messrs. A. D. Innes and Co., announce a new Church Magazine, well illustrated and of a popular character, to begin with the new year, to be named "The Minster." The Newbery House Magazine will be at the same time withdrawn. It is the purpose of "The Minster" to occupy a field which hitherto has been left vacant; inasmuch as none of the magazines of a distinctively religious character are primarily conducted for Churchmen. The proprietors intend to produce a magazine which will be interesting and popular, but will at the same treat the graver questions of the day which appeal directly to Churchmen in a weighty and effective manner. The January number will appear shortly before Christmas, and the contributors to this, the first number, include: the Archbishop of Canterbury, the Dean of St. Paul's, the Headmaster of Harrow (the Rev. J. E. C. Welldon, D.D.), Wilfrid Cripps, C.B., George Spottiswoode, Dayrell Trelawney, Sir Edwin Arnold, K.C.I.E., Sir Benjamin Baker, K.C.M.G., George Saintsbury, Corney Grain, Linley Sambourne, George Gissing, and James Payne.

